# Guanosine: a Neuromodulator with Therapeutic Potential in Brain Disorders

**DOI:** 10.14336/AD.2016.0208

**Published:** 2016-10-01

**Authors:** Débora Lanznaster, Tharine Dal-Cim, Tetsadê C. B. Piermartiri, Carla I. Tasca

**Affiliations:** ^1^Departamento de Bioquímica,; ^2^Programa de Pós-graduação em Neurociências, Centro de Ciências Biológicas, Universidade Federal de Santa Catarina-UFSC, Campus Trindade, 88040-900, Florianópolis, SC, Brazil; ^3^CAPES Foundation, Ministry of Education of Brazil, Brasília - DF 70040-020, Brazil

**Keywords:** guanosine, purines, neuromodulator, neuroprotection, neurotrophic effects, glutamate, adenosine

## Abstract

Guanosine is a purine nucleoside with important functions in cell metabolism and a protective role in response to degenerative diseases or injury. The past decade has seen major advances in identifying the modulatory role of extracellular action of guanosine in the central nervous system (CNS). Evidence from rodent and cell models show a number of neurotrophic and neuroprotective effects of guanosine preventing deleterious consequences of seizures, spinal cord injury, pain, mood disorders and aging-related diseases, such as ischemia, Parkinson’s and Alzheimer’s diseases. The present review describes the findings of *in vivo* and *in vitro* studies and offers an update of guanosine effects in the CNS. We address the protein targets for guanosine action and its interaction with glutamatergic and adenosinergic systems and with calcium-activated potassium channels. We also discuss the intracellular mechanisms modulated by guanosine preventing oxidative damage, mitochondrial dysfunction, inflammatory burden and modulation of glutamate transport. New and exciting avenues for future investigation into the protective effects of guanosine include characterization of a selective guanosine receptor. A better understanding of the neuromodulatory action of guanosine will allow the development of therapeutic approach to brain diseases.

Guanosine is an endogenous guanine nucleoside to which has been attributed several neuroprotective and neurotrophic effects. This review will describe the extracellular role of guanosine as an intercellular messenger in the Central Nervous System (CNS). We also introduce a brief historical overview of the purinergic system, centering in the guanine-based purinergic system.

## Purinergic system

Purines are a class of aromatic organic molecules essential for all cells that include adenine- and guanine-derivatives nitrogenous bases, as nucleotides with one or more phosphates (AMP, ADP, ATP and GMP, GDP, GTP, respectively) and related metabolites such as adenosine, inosine, adenine, hypoxanthine and guanosine, guanine, xanthine and uric acid.

Intracellular purines are primarily identified as structural constituents of nucleic acids, but they are also part of the structure of some coenzymes, and display roles as second messengers. Adenine-based nucleotides are well known for their fundamental intracellular role in the maintenance of energetic metabolism in the cells [1].

The adenine-based purines effects in different cell types have unfolded the role of purines in cell signaling. Important biological functions of adenosine were initially unraveled by Drury & Szent-Giorgy [2], demonstrating that adenosine is released to the extracellular space after heart ischemia, promoting a negative chronotropic effect to the heart and acting as a vasodilator of coronary vessels. The adenine-based triphosphorylated nucleotide, ATP, was also recognized as having extracellular effects [3] and in the 70’s Burnstock had demonstrated the concept of purinergic nerves and purinergic neurotransmission [4, 5]. From then on, adenosine and ATP have been recognized as intercellular messenger molecules and a pivotal role of these purinergic messengers in the CNS has been highlighted elsewhere [6-10].

Similarly to adenine derivatives, guanine-based purines have been firstly identified by their intracellular effects as modulators of G-proteins function. Guanine-nucleotide regulatory proteins, GTP binding proteins, or simply G-proteins have been identified as central actors in the signal transduction field, by coupling transmembrane protein receptors to intracellular effectors [11]. G-proteins activity modulation occurs via interaction with GDP in the basal state (associated in the p-loop of the alpha-subunit of heterotrimeric G-proteins), and with GTP in the activated state, when alpha-subunit dissociates from beta-gamma subunits [12]. Guanine nucleotides have also been shown to modulate the activity of small (low-molecular) monomeric G-proteins such as Ras, Rab Ef-Tu, and others [13]. To date, there is no evidence of interaction of guanosine to G-proteins.

As expected, guanine nucleotides and the nucleoside guanosine have also been shown to exert extracellular effects and a guanine-based purinergic system has been highlighted after innumerous findings demonstrating the extracellular actions of GTP, GMP and guanosine, most of them concerning CNS effects [14]. In their review, Souza and colleagues proposed and described the organization of the guanine-based purinergic system in the mammalian CNS and ever since it is accepted as an important intercellular messengers system compared to the adenine-based purinergic system.

In the present review, we will briefly discuss the guanine-based purinergic system and then we will focus on the extracellular effects of the guanine nucleoside guanosine.

## Guanine-based purinergic system

The extracellular effects of guanine-based purines or guanine derivatives have been primarily shown in the CNS, and these effects are relating to the modulation of the glutamatergic system, the main excitatory neurotransmission system in the brain [15]. The neurotransmitter glutamate exerts essential trophic effects in the CNS, but it may act as an endogenous toxin after brain injury after excessive release to the synaptic space, evoking a cascade of cellular death widely known as excitotoxicity. This harmful action of glutamate occurs mainly through activation of ionotropic glutamate receptors (iGluRs), namely N-methyl-D-aspartate (NMDA), Kainate (Ka) and alpha-amino-phosphonic acid (AMPA) receptors, but also of metabotropic glutamate receptors (mGluRs) [16]. However, the complete blockade of glutamate receptors activity is not beneficial to neural cells. Clinical trials blocking glutamate receptors, such as the use of dizocilpine (MK-801, an NMDA receptor antagonist) in traumatic brain injury and ischemia did not show an effective outcome, because this NMDA receptor blockade also prevents the necessary trophic wave of glutamate receptors activation after injury [17]. Therefore, molecules that may act as glutamatergic modulators without inhibiting glutamate physiological function have a fundamental importance in neuroprotection. In this scenario, guanine-based purines emerge as endogenous modulatory agents of glutamatergic transmission eliciting important interactions with glutamate receptors and transporters.

Binding studies initially performed by Sharif and Roberts [18] and then confirmed by other groups [19-23] showed that guanine derivatives displaced glutamate binding and analogs to its receptors in cell membrane preparations. Several studies from Ramirez G. and Souza D.O. laboratories were fundamental in order to show that this effect of guanine nucleotides did not rely on G-proteins interaction or its ability of reducing agonist binding to G-protein coupled receptors (GPCRs) when they are interacting with G-proteins [24-28]. This conclusion are further confirmed by the fact that GMP, which does not bind to G-proteins, is able to decrease glutamate binding to metabotropic and to ionotropic receptors that do not interact with G-proteins [29-34]. Guanine derivatives not only bind to glutamate receptors but they also abolished several glutamate-induced cell responses, in physiological [34-38] or in pathological situations [32, 38].

A seminal study from Souza and Ramirez [27] on guanine derivatives interaction with glutamate receptors in cellular membrane preparations was the first demonstration showing that there is a possible selectivity effect of guanine nucleotides in order to displace glutamate binding to its receptors, but not the nucleoside guanosine. However, we have found that some neurotrophic effects of guanosine are abolished by glutamate receptors antagonists [39].

In addition to modulating glutamate binding to its receptors, guanine derivatives modulate glutamate transport. Guanine derivatives are involved in the regulation of glutamate uptake into synaptic vesicles [40], suggesting a modulatory role of guanine-based purines on glutamate turnover. Several recent studies have shown that guanosine modulate glutamate transporters activity (see discussion below), although no study so far has demonstrated a direct interaction of guanosine with glutamate transporters.

Additional evidence of guanine-based purines importance in the extracellular space were obtained from studies showing that purine nucleosides are released after an ischemic injury and their levels are maintained elevated from 2 hours to 7 days [41]. Cultured astrocytes subjected to hypoxic or hypoglycemic situations also release purine nucleotides and extracellular levels of guanine derivatives may reach three-fold higher levels than adenine derivatives [42]. These evidences suggested that guanine-based purines might represent an endogenous restorative system activated after injury situations. Moreover, the extracellular presence of guanine derivatives was also identified in samples of human cerebrospinal fluid (CSF) [43].

**Figure 1.**
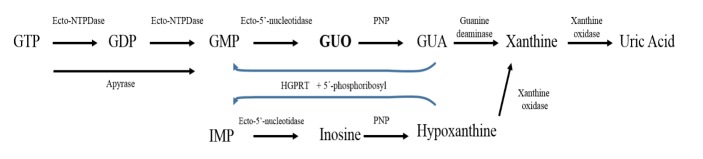
**Guanine-based purines catabolism**. GTP, GDP and GMP are hydrolyzed sequentially by nucleotidases (or ecto-nucleotidases, when produced extracellularly), generating guanosine (GUO). Ecto-NTPDase (or apyrase) metabolizes GTP and GDP to produce GMP. Guanosine is hydrolyzed by PNP generating the purine base guanine (GUA). By action of a guanine deaminase, guanine is converted to xanthine and sequentially to uric acid by action of a xanthine oxidase. The salvage purines pathway enzyme HGPRT produces GMP or IMP from condensation of GUA or hypoxanthine with 5’-phosphoribosyl, respectively (blue arrows). Ecto-NTPDase, ecto-nucleotide diphosphohydrolase; HGPRT, hypoxanthine-guanine phosphoribosyltransferase; PNP, purine nucleoside phosphorylase.

Extracellular nucleotides are hydrolyzed by a family of ecto-nucleotidases associated to the cell surface, the ecto-nucleoside triphosphate (ecto-NTPase) family [44]. The ecto-NTPases include the ecto-ATPase that hydrolyses ATP and GTP (and also pyrimidine nucleotides with less affinity) to ADP and GDP; the ecto-ATP-diphosphohydrolase or apyrase (ecto-NTPDase), that hydrolyses either ATP or GTP and ADP or GDP to AMP or GMP [45]; and the ecto-5’-nucleotidase that hydrolyses AMP or GMP to the nucleosides adenosine and guanosine [46] (Fig. 1). Thus, after brain injury, released nucleotides undergo hydrolysis and their respective nucleosides may display a protective effect. In addition to this evidence of guanine derivatives being released in pathological situations, our group showed that GTP is taken up and stored into brain synaptic vesicles, suggesting that this nucleotide may act as a neurotransmitter [47].

In conclusion, major findings regarding the guanine-based purinergic system are: (i) guanine nucleotides displace the binding of glutamate and analogs to metabotropic and ionotropic glutamate receptors; (ii) guanine nucleotides present a competitive pattern of antagonistic interaction with glutamate receptors; (iii) the effects of guanine-based purines do not rely only on G-proteins interaction; (iv) guanine-based purines are present in the extracellular space and their released may be increased under certain harmful conditions; (v) extracellular guanine nucleotides may be hydrolyzed to guanosine and increase the extracellular level of this nucleoside after brain damage; (vi) guanine-based purines modulate glutamate transporters (located at synaptic vesicles and at cellular membrane) activity; (vii) guanosine modulates glutamate transporters activity although it is uncertain whether it directly interacts with glutamate transporters.

This review discusses the extracellular roles of guanosine from *in vivo* and in *in vitro* experimental approaches and presents an update on guanosine effects and mechanisms of action as an intercellular messenger mainly in the CNS. Although the focus of this review is guanosine as a protective and trophic messenger in the CNS, some peripheral effects that help to further understand the mechanism of guanosine action are also discussed.

## Metabolism and distribution of exogenous Guanosine

Guanosine is a nucleoside that may act as a neuroprotective or retaliatory endogenous system. However to evaluate the effects of guanosine studies often use exogenous administration of this nucleoside. Hereafter, we will discuss the metabolism and distribution of exogenously administrated guanosine followed by the presentation of guanosine effects in *in vivo* models of brain disorders.

Acute intracerebroventricular (i.c.v.) administration of guanosine resulted in a significant and rapid (5 min after) increase of guanosine and its metabolites xanthine and uric acid in the cerebrospinal fluid (CSF), but did not affect hypoxanthine or others nucleotides and nucleosides CSF levels [48] indicating an *in vivo* breakdown of the administered guanosine. In fact, the enzyme purine nucleoside phosphorylase (PNP, which converts guanosine to guanine) and guanine deaminase (that converts irreversibly guanine to xanthine) were identified at brain membranes, and their activities may result in elevated levels of purines metabolites in the brain [49].

After systemic administration, guanosine levels rapidly increase in the CNS. Intraperitoneal (i.p.) administration of GMP or guanosine (7.5 mg/kg) increases guanosine CSF levels around two-fold and three-fold respectively, after 30 min [50], and i.p. guanosine administration increases guanosine and guanine levels analyzed in the spinal cord [51].

Evaluation of guanosine metabolism after sub-chronic guanosine administration (8 mg/kg, i.p.) in mice for 15 days induced increase in GDP and xanthine hippocampal levels, when analyzed 5 days after the last treatment (Lanznaster D. et al, unpublished data). In rats subjected to a treatment protocol where guanosine was added to the drinking water during 6 weeks presented elevated xanthine levels in CSF and plasma samples, and levels of adenosine and hypoxanthine were elevated in rats plasma [52]. These data confirm guanosine breakdown both at CNS and periphery, and guanosine-induced adenosine release, as showed before [49], might explain the increased adenosine levels in the rat plasma. More studies are necessary to clarify the distribution of guanosine by oral route, considering the effectiveness of oral administration of guanosine [53-58].

Guanosine distribution through tissues after systemic administration is reported in rats [51, 59]. In the first study, rats received guanosine (8 mg/kg - i.p.) and radioactivity peaked about 15 min after injection in the heart, kidney, liver and lungs. In the adipose tissue and CNS [^3^H]-guanosine concentration peaked about 30 minutes after injection. Further investigations on guanosine metabolism revealed that guanine was the major metabolic product in all sites, with over twice as much guanine compared to guanosine after 30 minutes [60] suggesting the occurrence of a rapid breakdown of the guanosine. In the second study, guanosine distribution and metabolism were demonstrated after different doses (2, 4, 8 and 16 mg/kg) in the presence of trace amount of [^3^H]guanosine, also given i.p. [59]. Radioactivity increased time- and dose-dependently in the plasma, reaching a plateau after 60 min. Guanosine and guanine levels were significantly higher in all analyzed tissues than the plasma, indicating a rapid distribution and accumulation at different organs including CNS. This study also demonstrated that plasmatic activity levels of the enzyme PNP were elevated, what might be associated to the rapid guanosine metabolism. Xanthine levels were higher at liver and kidneys, suggesting that these organs play an important role in the metabolism and possibly excretion of guanosine. This data is supported by a previous study, where [^3^H]guanosine given via intramuscular was primarily found at animals kidney [61].

Taken together, these data show that systemic administration of guanosine reaches central and peripheral nervous systems in order to exert its functions. Regarding guanosine metabolism, several studies confirm the rapid conversion into guanine, thus raising the question if the biological activity observed is directly dependent from guanosine [51, 59, 60]. Although there are no studies to date reporting the neuroprotective effect of guanine, only one *in vitro* study showed that guanosine (100 μM) but not guanine treatment increased cell proliferation of neural stem cells in culture [62], suggesting that, at least for neurotrophic effects, guanosine is the bioactive molecule.

## *In vivo* effects of Guanosine

### Neuroprotective effects of Guanosine

Several studies have shown the neuroprotective effect of guanosine in animal models of CNS disorders in both rats and mice, and hereafter we discuss these findings.

#### Seizures

Seizures are often related to an overstimulation of glutamatergic activity. Quinolinic acid (QA, an endogenous NMDA receptor agonist) is involved in epilepsy ethiology and induce seizures when it is exogenously administered in the rodent brain [15, 63, 64]. Acute administration of guanosine i.p. reduced QA-induced seizures about 50 - 70% [65-69], and guanosine neuroprotective effect is also observed when guanosine was administered via intracerebroventricular (i.c.v) [70] or orally [53, 55], showing that anticonvulsant effect of guanosine is effective regardless the route of administration. In a chronic treatment protocol, guanosine added at the drinking water during two weeks decreased seizures induced by QA and by α-dendrotoxin, a potassium channel blocker that promotes the endogenous release of neurotransmitters like glutamate [54, 71].

Guanosine prevents QA-induced seizures in a similar degree of the NMDA receptor antagonist, MK-801 [68]. Besides acting as an NMDAR agonist, QA modulates glutamate transport and guanosine was shown to counteract QA-induced decrease in glutamate uptake [55] and the increase in synaptosomal glutamate release [67], reinforcing the hypothesis that guanosine modulates glutamatergic system activity.

Other guanine-based purines have been shown anticonvulsant effect against QA, but it seems that this effect is dependent upon their breakdown to guanosine, once GMP anticonvulsant effect is abolished after treatment with the 5’-nucleotidase inhibitor alpha-beta-methylene-adenosine-5’-di-phosphate (AOPCP), that inhibits GMP breakdown to guanosine [66].

Guanosine also modulates changes in eletro-encephalographic (EEG) signals induced by QA i.c.v infusion. QA infusion disrupts a prominent basal theta (4-10 Hz) activity during peri-ictal periods and also promotes an increase in gamma (20-50 Hz) oscillations. These EEG alterations are counteracted by guanosine when seizures are successfully prevented [69].

In a recent study, by using a genetic model of absence epilepsy (WAG/Rij rats), guanosine reduced the number of spike-wave discharges related to abscence epileptic activity. This effect was independent of adenosinergyc system, as the anti-epileptic effect of guanosine is not altered by co-treatment with teophylline (a non-selective adenosine receptors antagonist) [72].

#### Ischemia

Brain ischemia is the major cause of disability worlwide, and the reduction in blood flow associated with ischemic events in the brain leads to a decrease in oxygen and glucose supplies in the affected area, resulting in cellular bioenergetics faillure followed by excitotoxicity and oxidative stress events [73].

Neuroprotective effect of guanosine was evaluated in several models of brain ischemia. In a perinatal hypoxia-ischemia (HI) model, neonatal rats (P7) were subjected to an unilateral occlusion of the common carotid artery and exposed to an hypoxic athmosphere (8% O2, 92% N2) for 1.5h, resulting in reduced glutamate uptake 3 to 5 days after the insult. Guanosine treatment immediately before, immediately after, 24h and 48h after HI recovered HI-induced reduction in glutamate uptake [74]. Following this study, the same group showed that the first guanosine administration 6h after HI also induced an increase in glutamate uptake [75]. Moreover, different protocols of guanosine treatment protected adult rats from neurological damages associated with unilateral middle cerebral artery occlusion (MCAO), improving gait disturbances and spontaneous activity, and reducing infarcted area [76-78].

Reduction in cerebral blood flow is a pathological feature of chronic cerebral hypoperfusion, which is associated with neurological vascular diseases and acounts for 10-50% of all dementias worldwide [79, 80]. An usefull model to study cerebral hypoperfusion is the permanent bilateral occlusion of common carotid arteries in adult rats, ensuing progressive and long-lasting neuronal damage and cognitive deficits [81-83]. Using this model, Ganzella and coworkers [57] found that oral guanosine treatment for two weeks after the hypoperfusion induction reverses the lost of pyramidal neurons and the increase in glial fibrillar acidic protein (GFAP) at hippocampal CA1 region, but has no effect over cognitive deficit induced by permanent bilateral occlusion of common carotid arteries.

Guanosine treatment also protects rats subjected to permanent cortical focal ischemia induced by thermocoagulation [84, 85]. This model leads to an impairment of the forelimb function and an increase in lipid peroxidation in the infarcted area, and guanosine treatment recovered these alterations. Furthermore, guanosine reduces cortical infarcted area by 40% and decreases neuronal degeneration. Guanosine also prevents the increase in reactive oxygen species (ROS) and reactive nitrogen species (RNS) levels, and increases the function and expression of important antioxidant defenses decreased by focal ischemia, like glutatione and superoxide dismutase (SOD). Regarding inflammatory pathways, guanosine reduces microglia activation induced by focal ischemia, and restores inflammatory mediators levels, like tumor necrosis factor (TNF-α), interferon-gamma (INF-γ) and interleukins, IL-1 IL-6 and IL-10 both in the CSF and infarcted area [85]. These neuroprotective effects are observed in a guanosine treatment protocol that initiates soon after thermocoagulation, pointing to a possible use of guanosine in clinical treatments performed immediately after ischemic damage.

#### Parkinson’s disease

Parkison’s disease (PD) is a neurodegenerative disorder characterized by massive dopaminergic neuronal death at substantia nigra pars compacta (SNc), causing motor symptoms like bradykynesia, rigidity and postural difficulties [86]. In a parkinsonism rodent model induced by administration of a proteasome inhibitor, guanosine decreases neuronal apoptotic cell death and increases dopaminergic neurons at SNc, accompanied by an improvement of motor symptoms (i.e. reduction of bradykinesia) [87].

Recently, a metabolomic analysis of PD-related alpha-synuclein A53T transgenic mice suggested that the interaction effect of aging and genotype disturbed only guanosine levels in the brain, amongst more than 200 metabolites analyzed. This study observed lower levels of guanosine in young A53T transgenic mice (3 month-old) compared with age-matched non-transgenic controls. There is no alteration of guanosine levels between young and old (18-month-old) non-transgenic mice. However, aged A53T transgenic mice showed increased guanosine levels compared to young transgenic mice. The authors suggest that increased guanosine levels in aged transgenic mice might represent a protective mechanism against neurodegeneration [88]. However, it is important to consider that these observations were obtained from the whole brain analysis, whereas neurodegeneration in PD patients and in this mouse model may be more restricted to basal ganglia structures and more specifically to the loss of dopaminergic nigrostriatal neurons [89]. Moreover, alterations in purines metabolism may be considered as biomarkers for PD diagnosis. Analyzed plasma samples from PD patients showed significantly reduced levels of uric acid, the end-product metabolite of purines catabolism (Fig. 1) [90].

#### Alzheimer’s disease

The possible neuroprotective effect of guanosine in a mouse model of Alzheimer’s disease is currently under investigation in our laboratory. Preliminary data indicates that guanosine (8 mg/kg, i.p.) treatment for two weeks prevents the increase in sodium-independent hippocampal glutamate uptake induced by i.c.v. infusion of β-amyloid_1-40_ peptide in mice (Lanznaster et al., unpublished data), pointing to a modulatory effect of guanosine against glutamatergic toxicity induced by β-amyloid.

#### Hepatic encephalopathy

Hepatic encephalopathy (HE) is a neurological condition associated with a cognitive impairment initiated by liver dysfunction, where ammonia is the major toxin. In animal models, the ammonia leads to an alteration in glutamate neurotransmission, increasing the levels of extracellular glutamate [91, 92]. The neuroprotective effect of guanosine was evaluated in rats subjected to bile duct ligation (BDL), an animal model of HE [93]. Guanosine (7.5 mg/kg, i.p.) treatment for 7 consecutive days from 2 weeks after surgery reverses BDL-induced cognitive impairment, without producing changes in ammonia levels. This HE model increases CSF glutamate levels and oxidative stress in the striatum and hippocampus, and guanosine restored most of these alterations, which may be related to the cognitive recovery induced by guanosine.

#### Sepsis

Sepsis induction performed by cecal ligation and perforation results in an increase in oxidative stress in the hippocampus, striatum, cerebellum and cerebral cortex. One single guanosine administration (8 mg/kg, i.p.) reduces lipid peroxidation induced by sepsis. Guanosine treatment for 10 consecutive days reduces cognitive impairment and depressive-like behavior induced by sepsis [94], suggesting that the neuroprotective effect of guanosine might be related to its ability to reduce lipid peroxidation in the brain.

#### Spinal cord injury

Guanosine administration was able to recover locomotor activity in rats subjected to a moderate spinal crush damage and a chronic traumatic spinal cord injury [95]. After spinal cord injury, remyelination of damaged area is critical for functional recovery [96-100]. Guanosine treatment increases bromo-deoxy-uridine (BrdU) incorporation in spinal cord slices, a marker for cell proliferation, atributable to an increase in the number of oligodendroglial progenitor cells. Increase in progenitor cells induced by guanosine is accompanied by the presence of mature oligodendrocytes at damaged area, allowing axonal remyelination and enhancing functional recovery [95, 101].

### Neurogenic effects of Guanosine

Some of guanosine therapeutic effects are due to its trophic actions, as guanosine induces increase in cell proliferation and neurogenesis. Active adult neurogenesis occurs in two areas of the brain: (i) the subgranular zone (SGZ) in the dentate gyrus of the hippocampus, and (ii) the subventricular zone (SVZ) of the lateral ventricles where neuroprogenitor/stem cells initially reside and proliferate prior to migration and differentiation [102, 103]. Systemic administration of guanosine for eight weeks (8 mg/kg) stimulates neuroprogenitors proliferation in the SVZ in a mice model of Parkinsonism [87]. The effect of guanosine treatment was accompanied by an increased number of fibroblast growth factor (FGF-2)-positive cells which is an important regulator of neuroprogenitor/stem cell proliferation, survival and differentiation [104]. Future studies might investigate if this proliferative effect of guanosine is followed by increased cell survival and differentiation.

### Antinociceptive effects of Guanosine

The antinociceptive effect of guanosine was demonstrated in nociception animal models, both in rats and mice. In a neuropathic pain model induced by chronic sciatic nerve constriction in rats, guanosine treatment reduced thermic hyperalgesia and motor deficit and prevented weight lost [105]. Guanosine reduces nociception in several pain models, as i.p. injection of acetic acid, formalin, glutamate or capsaicin. Guanosine also inhibits nociception induced by non-NMDA receptor agonists administered via intrathecal. Mice treated with guanosine showed increased latency when exposed to the hot plate test [56]. Nociceptive behavior associated with the hot plate test (i.e., jumping and liking the hind paws) is considered to be organized supraspinally [106]. Taken togheter, these results suggest that systemic guanosine acts at central structures, once guanosine treatment increases mice latency at the hot plate test and inhibit nociception induced by central administration (i.e. at spinal cord) of nociceptive substances [56]. In agreement with these findings, previous studies showed that guanosine levels on central structures rises after minutes of intraperitoneal administration [59, 60, 105]. Moreover, central (i.c.v.) administration of guanosine in mice presented antinociceptive effect against chemical (glutamate- and capsaicin-induced liking behavior) and thermal (tail flick and hot plate) nociceptive models [107], reinforcing the hypothesis of a CNS action of guanosine in order to promote its antinociceptive effect.

### Guanosine effects on neuropsychiatric disorders

#### Anxiety

Guanosine administration promoted anxiolytic-like behavior in mice and rats. Guanosine added to the mice drinking water during two weeks increased head-dips and crossings in the hole-board behavior test when compared to the effect observed with the administration of Diazepam, a classical anxiolytic drug [54, 108]. Administration of guanosine i.p. in rats increased time spent in the open arms of the elevated plus-maze behavior test also compared to Diazepam, confirming an anxiolytic-like behavior induced by guanosine [109].

#### Depression

Depression is a leading cause of disability worldwide. Recently, there is increasing evidence supporting a role for glutamate transmission in the etiology and treatment of depression and the use of compounds that modulate glutamatergic system such as Ketamine, has demonstrated to produce a rapid-acting antidepressant effect [110]. Guanosine treatment has been show to present similar biological effects of ketamine ensuing fast antidepressant effect and possible modulation of NMDA receptors. A single oral administration of guanosine (0.05 - 5 mg/kg) in mice resulted in antidepressant-like activity in the forced swimming and tail suspension tests [111]. To date there are no studies of chronic use of guanosine in depression. Increasing adult neurogenesis is a promising line of research against depression (for a revision see [112] and studies have suggested that neurotrophins are involved in the neurogenic action of antidepressants [113]. Guanosine neurotrophic effect and further activation of intracellular pathways may enhance neuroplasticity and neurogenesis contributing to a long-term sustained improvement of antidepressant-like effect in rodents.

Recently, several studies have associated mood disorders with stressful lifetime events (for a revision see [114]). Mice subjected to acute restraint stress (a 7 h-immobilization period, restraining every physical movement) presented an increase in immobility time, a parameter of depressive-like behavior analyzed in the forced swimming test. A single dose of guanosine (5 mg/kg, p.o.) reversed this depressive-like behavior and decreased stress-induced increase in hippocampal TBARS. Guanosine also prevented alterations induced by stress in the antioxidant enzymes catalase, glutathione peroxidase and glutathione reductase, confirming guanosine ability to modulate antioxidant system in the brain [58].

#### Schizophrenia

Using a mouse model of schizophrenia with administration of MK-801, Tort el al. [115] demonstrated some anti-psychotic effect of guanosine. MK-801 is an uncompetitive antagonist of the NMDA receptor that induces hyperlocomotion in mice. Approximately 20 min after i.p. MK-801 administration, mice presented an increase in their locomotor activity. Guanosine pretreatment (30 min before MK-801) decreased about 60% of this altered locomotor behavior. Authors also showed that guanosine did not change the hyperlocomotion induced by caffeine or amphetamine, indicating a direct guanosine action over the glutamatergic transmission in this model.

**Table 1:** Summary of Guanosine *in vivo* and *in vitro* effects

*In vivo* effects	Experimental approach	References
*Neuroprotection*		
Prevented seizures and EEG changes induced by quinolinic acid	Mouse	[53]; [54]; [55]; [69]
Improved motor disturbances and neural damage associated with ischemia/hypoxia models	Rat	[74]; [76]; [52]; [84]
Reduced motor deficit and dopaminergic neuronal loss in a parkinsonism model	Mouse	[87]
Reversed cognitive impairment and oxidative parameters induced by a model of hepatic encephalopathy	Rat	[93]
Inhibited TBARS increase and cognitive deficit associated with sepsis	Rat	[94]
Increased motor recovery, proliferation of progenitor cells and remyelination in spinal crush model	Rat	[95]; [101]
*Neurogenic effects*		
Stimulated neuroprogenitors proliferation in the SVZ and increased number of FGF-2-positive cells	Mouse	[87]
*Antinociception*		
Prevented nociception induced by acetic acid (i.p.) and by formalin, capsaicin or glutamate (i.pl.); increased latency at hot plate test	Mouse	[105]
Reduced thermic hyperalgesia and motor deficit associated with sciatic nerve constriction	Rat	[70]
*Anxiolytic*		
Increased head-dips and crossings in the hole-board model	Mouse	[54]
Increased time spent in the open arms of the elevated plus-maze task	Rat	[109]
*Antidepressant*		
Reduced immobility time in forced-swimming and tail suspension tests	Mouse	[58]; [111]
***In vitro* effects**		
*Neuroprotective*		
Prevented the reduction of glutamate uptake induced by ischemia or glucose deprivation	Cortical slices	[120]
	Hippocampal slices	[121]; [122]; [124]
	C6 astroglial cells	[125]
Protected from glutamate toxicity by reducing iNOS and oxidative stress	Hippocampal slices	[126]; [127]
	HT22 cells	[128]
Reduced oxidative damage by increasing antioxidant enzymes and HO-1 expression	SH-SY5Y cells	[129]
	C6 astroglial cells	[130]
Prevented increase in proinflammatory mediators induced by ischemia, oxidative damage or inflammatory agents	Hippocampal slices	[124]
	C6 astroglial cells	[130]
	Cultured mouse microglia	[136]
Protected from apoptosis induced by staurosporine, Aβ and MPP+	Culture rat astrocytes	[158]
	SH-SY5Y cells	[132]; [134]
Inhibited oxidative damage and apoptosis induced by Aβ oligomers	SH-SY5Y cells	[135]
*Neurotrophic*		
Induced cell proliferation, synthesis and release of FGF-2 and NGF	Cultured rat astrocytes	[42]; [140]; [141]; [142]
Promoted neurite outgrowth	PC12 cells	[143]; [144]
Altered laminin and fibronectin from punctual to fibrillar organization	Cultured cerebellar astrocytes	[145]
Increased the number of neurons	Cultured cerebellar neurons	[39]
Increased cell proliferation and BNDF mRNA levels	Neural stem cells	[62]

Abbreviations: Aβ, amyloid-beta peptide; BDNF, brain-derived neurotrophic factor; EEG, electroencephalogram; FGF-2, fibroblast growth factor-2; HO-1, heme oxigenase 1; iNOS, inducible nitric oxide synthase; i.p.: intraperitoneal; i.pl.: intraplantar; MPP+, 1-methyl-4-phenylpyridinium; NGF, nerve growth factor; TBARS, thiobarbituric acid reactive substances; SVZ, subventricular zone.

## *In vitro* effects of Guanosine

### Neuroprotective effects of Guanosine

#### Ischemia

*In vitro* studies are a useful tool in order to elucidate the mechanisms of neuroprotective effects of guanosine. High levels of guanine-based purines were found during and after hypoxia or hypoglycemia, mainly guanosine [41, 42]. Moreover, the evidence that astrocytes exposed to hypoxic or low glucose environment increases guanosine levels in the extracellular space [116] prompted researchers to assess the neuroprotective role of guanosine in in vitro ischemic models.

Oxygen glucose deprivation (OGD) in brain slices is an *in vitro* ischemia model largely used in the literature to study putative neuroprotective agents [117, 118]. In hippocampal slices, where the neuroprotective effect is a resultant from interactions between neurons and glial cells, guanosine promoted neuroprotection against OGD when added to the re-oxygenation period [119].

The neuroprotective effects of guanosine may be related to its ability of stimulating glutamate uptake in situations of ischemic damage, as demonstrated in cortical slices subjected to OGD [120]. This finding was further confirmed from our group by demonstrating that modulation of glutamate uptake by guanosine was related to its mechanism of neuroprotection in rat hippocampal slices subjected to OGD [121]. Another study found that guanosine increased glutamate uptake in hippocampal slices from young rats (10 days) but it did not show any changes in hippocampal slices from adult rat slices subjected to OGD [122]. This discrepancy may be due to protocols differences. While we used a protocol of 15 min of OGD followed by 2 hours of re-oxygenation [121], Thomazi and colleagues [122] used a protocol of 1 hour of OGD followed by 1 or 3 hours of re-oxygenation. It is important to note that shorter OGD protocols are more compatible with pathological ischemic situation in humans.

Other effects of guanosine treatment in hippocampal slices subject to OGD include antioxidant effects by reducing oxidative parameters (i.e ROS production) and preventing mitochondrial membrane depolarization in CA1 region of hippocampal slices subject to OGD. Moreover, guanosine regulates inflammation by inhibiting p65 (active subunit of NF-κB transcription factor) translocation to the nucleus and reducing inducible Nitric Oxide Synthase expression [123, 124]. Recent findings demonstrated that guanosine reduces nitric oxide (NO) levels and displays similar protection to neuronal NOS (nNOS) or iNOS inhibitors against OGD (Thomaz, Dal-Cim, Tasca et al., unpublished data), suggesting a mechanism of NOS inhibition involved in the neuroprotection promoted by guanosine against ischemia.

**Figure 2.**
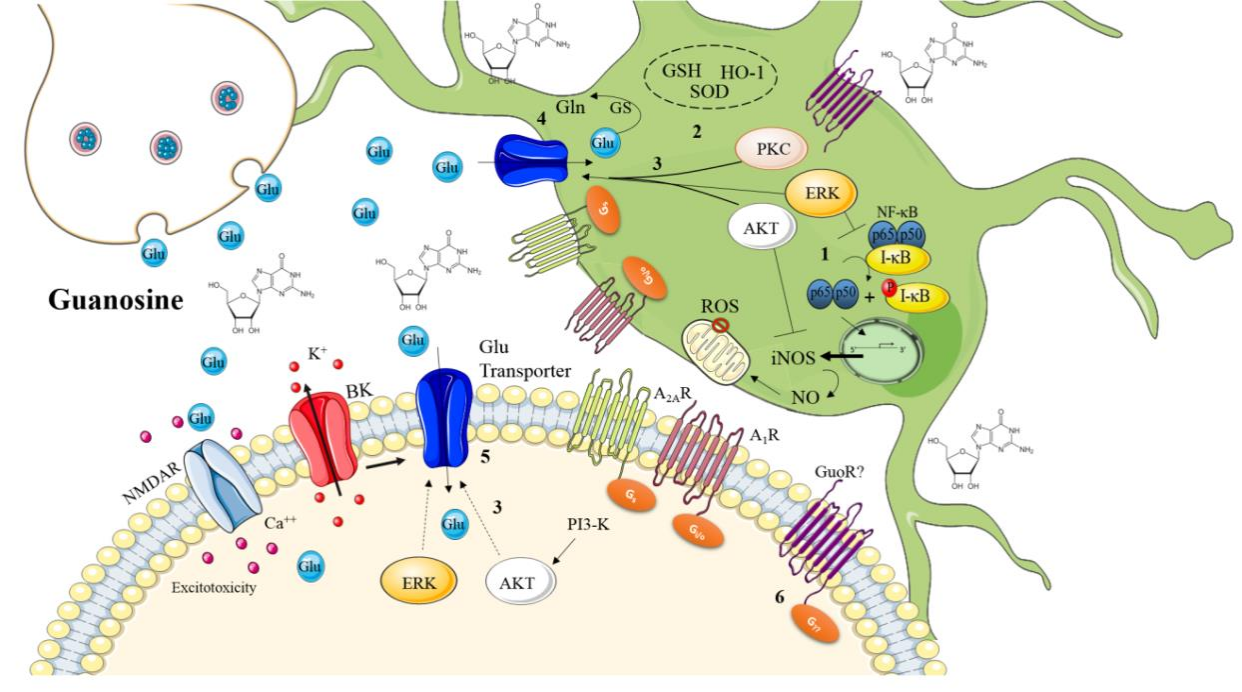
**Overview of the main mechanisms involved in the neuroprotective effects of guanosine**. Guanosine promotes neuroprotection through reduction of reactive oxygen species levels (ROS) by inhibition of nuclear factor kappa B (NF-κB) activation via MAPK/ERK and by preventing iNOS induction (1) [124]. Guanosine also counteracts ROS production by increasing antioxidant defenses [i.e. superoxide dismutase (SOD) activity and glutathione (GSH) and Heme-oxygenase (HO-1) levels] (2) [58, 84, 129, 130, 137]. Activation of PI3K/Akt, PKC and MAPK/ERK by guanosine leads to stimulation of glutamate transporters activity (3) [124-126]. Guanosine recovers glutamate transporters functionality and increases glutamine synthetase (GS) activity, thus reducing extracellular levels of glutamate and protecting from glutamate excitotoxicity (4) [152]. The inhibition of calcium-dependent (big) conductance potassium (BK) channels and activation of A_2A_R inhibits guanosine-induced increase in glutamate uptake (5) [124]. Guanosine promotes cell viability recovery by modulation of BK channels, A_1_R and A_2A_R [121, 124, 129]. A specific binding site for guanosine was identified as a putative GPCR (or GPR23), but this “guanosine receptor” (GuoR) was not yet fully characterized and its involvement in the neuroprotective effects of guanosine was not evaluated (6) [149, 150]. Figure designed using images from www.servier.com/Powerpoint-image-bank.

Cells lineages and primary cells cultures subjected to ischemic damage were also used in order to evaluate guanosine-induced neuroprotection. Guanosine was effective in preventing cell death induced by OGD in a neuroblastoma lineage, SH-SY5Y cells [76]. In a model of glucose deprivation in an astrocytoma cell lineage (C6 astroglial cells) guanosine increased glutamate uptake, expression of neuronal glutamate transporter EAAC1 and glutamine synthase activity, resulting in neuroprotection [125]. However, in primary cultures of cortical astrocytes, we have observed that guanosine protects from OGD by increasing glutamate uptake without altering the protein levels of GLT-1 transporter - the most effective glutamate transporter present in astroglial cells (Dal-Cim et al, unpublished results). Further studies, including the evaluation of expression or distribution of glutamate transporters might help to clarify the mechanisms evoked by guanosine in the modulation of glutamate transport in ischemic situations.

#### In vitro glutamate challenge

Evaluation of putative guanosine protection against glutamatergic excitotoxicity *in vitro* demonstrated that guanosine (100 µM) prevents glutamate damage to hippocampal slices by decreasing glutamate release and preventing iNOS induction [126]. Guanosine treatment was also able to attenuate glutamate-induced increased ROS production and decrease glutamate uptake in brain slices from adult rats [127]. Glutamate toxicity induced oxidative damage in the neuroblastoma cells HT22 and treatment of these cells with cGMP showed that the metabolites GMP and GUO are more effective in affording protection that cGMP. In the same study, it was demonstrated that guanosine protected from glutamate toxicity by increasing the levels of the cystine/glutamate antiporter system (Xc^-^), which is involved in maintaining the intracellular cysteine levels for glutathione synthesis [128].

These studies evaluating the role of guanosine against ischemic damage and glutamate toxicity added key information about the intracellular mechanisms evoked by guanosine and its ability to counteract events involved in neurodegeneration, as clearance of glutamate from the extracellular space, reduction of inflammation, activation of antioxidant defenses, and maintenance of mitochondria bioenergetics.

#### Mitochondrial stress

Using an *in vitro* protocol that evoked mitochondrial activity disruption induced by blockade of mitochondrial complexes I and V activity in SH-SY5Y neuroblastoma cells, we have shown that guanosine (at 1 mM for 24 hours treatment) can afford cytoprotection through induction of the antioxidant enzyme heme-oxygenase-1 [129]. Heme-oxygenase-1 was also involved in ability of guanosine to counteract oxidative and nitrosative stress and pro-inflammatory cytokines increase in C6 astroglial cells treated with an inhibitor of complex IV (i.e. azide) [130].

#### Parkinson’s disease

An *in vitro* model to study the mechanisms of cell death associated with Parkinson’s disease was assessed by using MPP^+^ (1-methyl-4-phenyl pyridinium), the active metabolite of the neurotoxin 1-methyl-4-phenyl-1,2,3,6-tetrahydropyridine (MPTP) that accumulates in the mitochondria and inhibits complex I activity and ultimately causes cell death [131]. Guanosine reverted apoptosis as assessed by DNA fragmentation and caspase-3 activity induced by MPP^+^ in SH-SY5Y neuroblastoma cells [132]. However, in another *in vitro* model of Parkinson’s disease where C6 astroglial cells are exposed to 6-hydroxidopamine, guanosine treatment was not able to reduce apoptosis levels assessed by apoptotic nuclei and oligonucleosome formation, despite promoting an improvement in reductive capacity of the cells [133].

#### Alzheimer’s disease and neuroinflammation

Few studies evaluated the neuroprotective effects of guanosine in *in vitro* models for Alzheimer’s disease by using β-amyloid peptides or also by inducing neuroinflammation. Treatment of SH-SY5Y neuroblastoma cells with guanosine protected cells against β-amyloid-induced apoptosis and ROS production [134, 135]. Guanosine also prevented increased β-secretase activity and increased β-amyloid_1-42_ levels induced by oxidative stress in SH-SY5Y cells [135].

In microglial cells exposed to β-amyloid_1-42_ guanosine prevented the expression and functionality of CD40 receptor (cell receptors associated with inflammatory events) by counteracting interleukin-6 (IL-6) production induced by pro-inflammatory agents such as TNF-α [136]. A recent study showed that guanosine prevented lipopolysaccharide (LPS)-induced inflammatory and oxidative damage in hippocampal astrocytes in culture and decreased pro-inflammatory levels of TNF- α and NF-κB by heme-oxygenase-1 induction [137].

As discussed above, guanosine displays protective role in *in vitro* protocols of glutamate challenge, mitochondrial stress, models of ischemia, Parkinson’s and Alzheimer’s diseases and neuroinflammation. From these studies can be stated that the mechanism of guanosine protection against neurodegeneration are related to its ability of modulating the glutamate transport, counteracting oxidative stress, preventing inflammatory damage, thus culminating in prevention from apoptosis. Fig. 2 presents evidence of neuroprotective mechanisms mediated by guanosine.

### Neurotrophic effects of Guanosine

**Figure 3.**
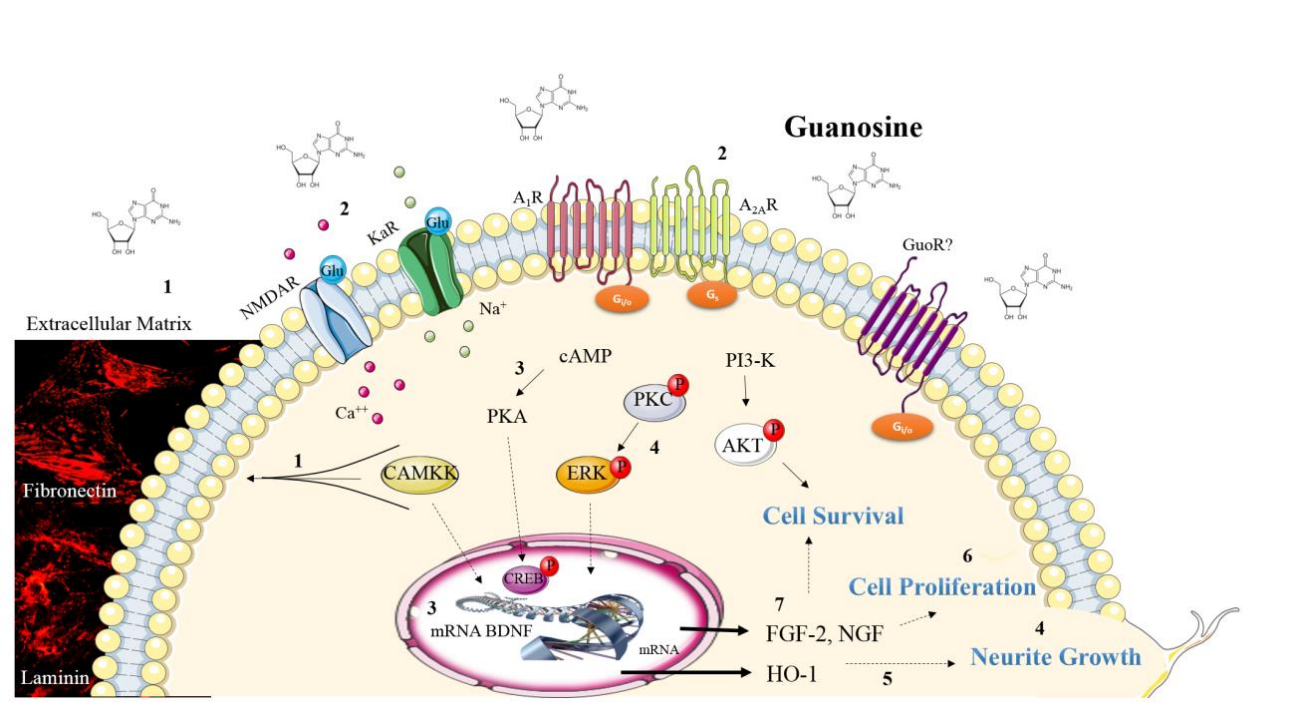
**Schematic illustration of the neurotrophic effects of guanosine**. In astrocytes cerebellar cultures guanosine promotes the reorganization of extracellular matrix proteins fibronectin and laminin (photomicrographs from Decker H. and colleagues [145]) via CaMKII, PKA, MAPK/ERK, PKC and PI3K/AKT activation (1) [145]. Guanosine also increases the number of cerebellar neurons in culture (or in coculture with astrocytes) by activation of these kinases. This guanosine neurotrophic effect involves A_2A_R activation and it is also dependent on NMDAR and Kainate receptors activation (2) [39]. In neural stem cells guanosine increases intracellular cAMP, CREB phosphorylation and BDNF mRNA levels (3) [62]. Guanosine promotes neurite outgrowth in cerebellar neurons culture by PKC activation (4) [143] and in PC12 by heme-oxygenase (HO-1) induction (5) [144]. In cultured astrocytes, guanosine promotes cellular proliferation (6) [116] and synthesis and release of neurotrophic factors, as FGF-2 and NGF (7) [141]. These neurotrophic effects of guanosine may be involved in cell survival. Figure designed using images from www.servier.com/Powerpoint-image-bank.

In the CNS, extracellular guanosine stimulates trophic effects on astrocytes and neurons [116, 138, 139]. Guanosine neurotrophic effects are depicted in Fig. 3.

The neurotrophic effect of guanosine treatment (300 µM for 24 hours) in increasing cell proliferation on cultured astrocytes was mediated by guanosine-induced adenosine release [116]. Guanosine also stimulates cultured astrocytic cells to increase synthesis and release of neurotrophic factors such as FGF-2 and neuron growth factor (NGF) [140, 141]. In pheochromocitoma (PC12) cells, guanosine treatment (300 µM for 48 hours) was able to enhance NGF-induced neurite arborization outgrowth [141, 142]. In fact, guanosine (500 µM) have been show to induce cellular protection through neurite arborization outgrowth in cultured cerebellar neurons and in PC12 cells subjected to hypoxia [143].

Treatment of PC12 cells with guanosine (300 µM) plus NGF promoting neurite outgrowth involved the induction of the antioxidant enzyme heme-oxygenase-1 and increased intracellular levels of cGMP [144]. These results showed that guanosine elicits an antioxidant response in these cells at the same concentration range (micromolar) it evokes neurotrophic and neuroprotective effects *in vitro*, suggesting these mechanisms may be interconnected.

The trophic effects of guanosine were also evaluated in neurons cocultured with astrocytes pretreated with guanosine (100 µM for 24 hours) in order to address a guanosine role on neuron-astrocytes interaction, a highly dynamic and reciprocal process. We observed that guanosine increased the number of cerebellar neurons in a neuron-astrocyte coculture and this effect was attributed to an action of guanosine modulating the extracellular matrix proteins, such as laminin and fibronectin organization [145]. Additionally, in a cerebellar neuronal culture treated with guanosine it was observed an increase in neurons number in culture. Similar results were observed when cerebellar neurons were cultured in a guanosine-treated astrocytic-derived conditioned medium [39]. Therefore, even a direct effect on neurons or via soluble factors released by astrocytes, guanosine promoted increased adhesion of neurons in culture. These guanosine effects may have an important role in neuronal migration and cell proliferation. Indeed, in neural stem cells culture from the SVZ, guanosine treatment (100 μM) was able to increase cell proliferation. The effect of guanosine was accompanied by increased expression of brain derived neurotrophic factor (BDNF) [62].

Guanosine neuroprotective effects observed in *in vitro* experimental approaches of neurotoxicity and guanosine neurotrophic effects cells cultures are summarized in Table 1.

## Guanosine-evoked cell signaling

In this section, we discuss the putative interaction sites for guanosine in cellular membranes and the intracellular signaling pathways involved in the biological effects of guanosine.

### Putative protein targets

There is still not a clear definition of a target receptorial protein specific to guanosine, since the “guanosine receptor” has not yet been identified. However, selective binding sites for guanosine were already demonstrated in rat brain membranes [146, 147]. Incubation of [^3^H]guanosine with total rat brain membranes preparations led to the identification of a single high affinity binding site for guanosine, with a dissociation constant (*Kd*) of 95.4 ± 11.9 nM and an apparent number of maximmal binding sites (Bmax) of 0.57 ± 0.03 pmol/mg protein. Both association and dissociation kinetics were rapid, which is characteristic of natural compounds binding to theirs receptors [146]. Other guanine-based purines and guanosine metabolites were not potent displacers of guanosine binding, neither did adenosine or the non-selective adenosine receptors antagonists caffeine and theophylline [147]. Drugs that inhibit the purine transporters systems were also investigated, since guanosine could bind to these proteins. Nitrobenzylthioinosine, an inhibitor of equilibrative nucleosides transporter, showed a small but no significant displacement at 10 µM, and nitrobenzylthioguanosine (a nucleosides transporter inhibitor) and propentofylline (adenosine reuptake inhibitor) had any effect on guanosine binding. Incubation of rat brain membranes with pertussis toxin (PTX), an inhibitor of Gα_i_ family proteins, reduced guanosine binding in 45% [147]. Additionally, by using a novel GTP-binding assay Volpini and coworkers identified a specific GPCR activated by guanosine that is different from the well-characterized adenosine receptors [148]. Taken togheter, these data point to the existence of a selective and putative guanosine receptor in rat brain membranes, although this protein was not isolated, sequenced, cloned and consequently there are no studies on structural prediction.

More recently, the receptor GPR23 is as a new candidate suggested for guanosine.GPR23 is one receptor for lysophosphatidic acid (LPA), identified as the LPA4 receptor. A communication from Di Liberto et al., [149] reported that guanosine reduces cell proliferation in a glioma cell line (U87), and the silencing of GPR23 decreased this effect. In the other hand, increasing GPR23 expression also increased guanosine antiproliferative effect. Radioligand binding assays revealed that overexpression of GPR23 increase guanosine binding to membrane fractions, and that both LPA and guanine were 10 times less effective than guanosine in displacing [^3^H]-guanosine binding to GPR23. Another meeting communication reported GPR23 expression and [^3^H]-guanosine binding in different brain areas and they found that cerebral cortex has the higher GPR23 expression and the maximal [^3^H]guanosine affinity binding site. Affinity binding site rank order for all tested areas was cortex > hippocampus > striatum > spinal cord [150]. Together these data suggest that GPR23 may represent a membrane target for guanosine, without discarding the possibility of guanosine interaction with other membrane proteins. Hereafter, we will discuss other putative target sites to guanosine interaction.

#### Glutamate transporters or receptors

Guanosine ability to modulate glutamatergic system was demonstrated by different groups, as previously discussed. Guanine nucleotides (namely GTP, GDP and GMP) have been shown to act as antagonist of glutamate receptors (for review see [14, 25] however guanosine had no effect on glutamate and analogs binding to glutamate receptors [54, 151].

Since guanosine increases glutamate uptake in different models reviewed here, glutamate transporters could represent a target for guanosine interaction. We have first identified the effect of guanosine on promoting glutamate uptake [121, 124] and reducing glutamate release [126] through intracellular signalling pathways (see discussion in the next section), suggesting that this glutamatergic modulation is secondary to an intracellular signalling pathway activated by guanosine. However, it also appears that guanosine interacts directly with glutamate transporters, once results from our laboratory showed that the presence of synthetic glutamate transporters inhibitors abolished the reduction of glutamate release promoted by guanosine [152]. To date, to the best of our knowledge, studies evaluating a direct interaction of guanosine with glutamate transporters were not reported.

#### Adenosine receptors

Guanosine effect over adenosinergic system is controversial. Caffeine, an adenosine receptor antagonist, reversed the anxiolytic-like behavior induced by guanosine in rats [109], but caffeine failed to inhibit guanosine antinociceptive effect against capsaicin i.pl. [107], and the anticonvulsant effect of guanosine on QA-induced seizures in mice [53]. Furthermore, caffeine had no effect on guanosine binding to rat brain membrane preparations [147]. Regarding the effect of selective adenosine receptors antagonists, DPCPX an A_1_R antagonist, inhibited guanosine antinociceptive effect in the capsaicin-induced pain model, but the same effect was not found when treating with SCH58261 an A_2A_R antagonist [56].

Trophic effects of guanosine on cultured cerebellar neurons had suggested an interaction with adenosine A_2A_R, since the antagonist ZM241385 abolished guanosine-induced increase in neuronal adhesion [39].

An *in vitro* evaluation of guanosine cytoprotective effect in a human neuroblastoma cell line (SH-SY5Y) subjected to mitochondrial oxidative stress was abolished by both adenosine A_1_R and A_2A_R antagonists (DPCPX and ZM241385, respectively)[129]. We also demonstrated that A_1_R mediates the neuroprotective effect of guanosine in hippocampal slices subjected to OGD, as DPCPX reversed guanosine-induced decrease in ROS formation and mitochondrial membrane potential, although DPCPX did not interfere with guanosine effect on glutamate uptake. The effect of guanosine of recovering glutamate uptake impairment caused by OGD was blocked by PTX (showing an interaction with a GPCR) and by the activation of A_2A_R by its agonist CGS21680. Thus, adenosine A_2A_R activation, but not the blockade with the antagonist ZM241385, inhibited guanosine-induced neuroprotective effect observed on cellular viability and glutamate uptake [124]. Since we observed that A_1_R blockade or A_2A_R activation can reverse guanosine-evoked neuroprotective effect, we hypothesized that guanosine effect may involve an interaction with A_1_R-A_2A_R oligomers, which are known to associate and interact in an antagonistic manner [153-155]. A possible interaction of guanosine with A_1_R-A_2A_R oligomers is currently under investigation in our laboratory.

#### Potassium channels

Participation of potassium (K^+^) channels activity on guanosine-induced effects was also assessed following the observation that guanosine was able to modulate K^+^ channels activity and expression. In cultured rat cortical astrocytes, chronic exposition (48h) to guanosine in high micromolar levels (500 µM) induced an increase in activity and expression of functional inward rectifier K^+^ channels [156]. Afterwards, we have shown that neuroprotective effect of guanosine may depend on K^+^ channels interaction, since charybdotoxin, an inhibitor of the large (big) conductance Ca^2+^-activated K^+^ channels (BK), abolished guanosine-induced increase on cellular viability in hippocampal slices subjected to OGD and in SH-SY5Y cells subjected to mitochondrial damage [121, 129]. This effect seems to be dependent on BK channels activity only, because glibenclamide, inhibitor of ATP-sensitive K^+^ channels, or apamin inhibitor of small conductance Ca^2^^+^-activated K^+^ (SK) channels, had no effect on guanosine-promoted neuroprotection, pointing to a selective effect on BK channels. BK inhibition also abolished guanosine effect of recovering glutamate uptake decrease in hippocampal slices subjected to OGD [121].

Whole cell patch clamp performed in HEK293 cells transiently transfected with the functional α-subunit of BK channels showed that guanosine promoted K^+^ conductance that was inhibited by the BK inhibitor iberiotoxin. Co-transfection of BK regulatory β-subunit did not modify the K^+^ conductance induced by guanosine, suggesting that guanosine may interact with the functional α-subunit of BK channels. Also, guanosine had no effect on the conductance of small conductance Ca^2+^-activated K^+^ channel (SK) channels [157], suggesting a selective interaction of guanosine with BK.

In conclusion, although there are reports claiming the existence of a selective guanosine receptor, several studies also demonstrated other putative interaction targets for guanosine, as glutamate transporters, adenosine receptors and potassium channels. To date, there is still puzzling information about guanosine selectivity to a protein target, what supports the idea that this nucleoside might act as a multi-target neuromodulator.

### Intracellular signaling pathways

#### Modulation of glutamate transport

Guanosine effect on modulation of glutamate transporter activity has an important neuroprotective mechanism against excitotoxicity events. To understand the mechanism by which guanosine stimulates glutamate transport, our group investigated the intracellular pathways related to the effect of guanosine in hippocampal slices subjected to glutamatergic excitotoxicity. We found that guanosine prevented glutamate release after glutamate challenge through activation of the phosphatidylinositol-3 protein kinase (PI3K) and protein kinase B (Akt) pathway [126]. Additionally, we demonstrated that guanosine stimulated glutamate uptake in hippocampal slices subjected to OGD through activation of the PI3K/Akt and mitogen-activated protein kinase/extracellular-regulated kinase (MAPK/ERK) [124, 126]. It has also been shown that guanosine stimulated glutamate uptake in C6 astroglial cells deprived of glucose by activation of PI3K/Akt, MAPK/ERK, protein kinase C (PKC) and p38^MAPK^ pathways [125]. Recent findings from our group indicated that guanosine stimulates glutamate uptake in astrocytic cells derived from rat cortex via modulation of MAPK/ERK, PKC and this effect did not involve increased GLT-1 levels but appears to increase its availability in astrocytes cellular membrane (Dal-Cim, unpublished data). Together, these studies conclude that the guanosine effect on glutamate transporters is dependent of signaling pathways activation, which ultimately could be modulating the activity, expression or availability of these transporters in the cell membrane.

#### Neuroprotection

Guanosine-induced cell viability also activates several signaling pathways including PKC, protein kinase A (PKA), MAPK/ERK and PI3K to protect hippocampal slices subject to OGD [119]. Guanosine increased activation and expression of Akt to protect astrocytes in culture against staurosporine (an apoptosis-inducing agent) [158], and decreased apoptosis induced by β-amyloid in cultured human neuroblastoma through the activation of PI3K and increased phospho-Akt [134]. Protection of guanosine against the blockade of mitochondrial complex I and V-induced oxidative damage involved activation of PI3K and increased activation of downstream targets Akt and GSK3β [129].

Guanosine also reduced p38^MAPK^ and Jun Kinase (JNK) induced by 6-OHDA and increased Akt phosphorylation and anti-apoptotic Bcl-2 protein expression in SH-SY5Y cells [159].

In summary, the PI3K/Akt pathway seems to be required for neuroprotection evoked by guanosine against ischemia [119], apoptosis [134, 158] and oxidative damage [129].

#### Inflammation

In microglia cell cultures, guanosine reduces inflammation and expression of pro-inflammatory proteins, an effect associated with the activation of PI3K pathway [136]. In hippocampal slices subject to OGD guanosine prevented NF-κB activation through MAPK/ERK and inhibited iNOS induction by PI3K and MAPK/ERK [124].

#### Antidepressant-like effect

Most of *in vivo* studies do not explore the signaling pathways involved in guanosine mechanism of action. However, Bettio and coworkers [111] have demonstrated that guanosine produces antidepressant-like effect in mice by activation of PI3K/Akt. The authors also demonstrated that rapamycin, an inhibitor of mammalian target of rapamycin (mTOR) prevent the antidepressant-like effect of guanosine, demonstrating for the first time the involvement of mTOR in the effects of guanosine.

#### Trophic effects

The intracellular signaling pathways by which guanosine promotes trophic effects has also been scarcely investigated. Guanosine protected primary cerebellar neurons culture from hypoxia and promoted neurite outgrowth by activation of PCK-related kinase-1 (PRK1) [143]. PRK1 is a member of PKC superfamily and is activated by interacting with the Rho and Rac families of small G-proteins and arachidonic acid [160]. In neurons, PRK1 appears to be involved in neuronal differentiation [161].

Guanosine promoted neural stem cells proliferation by stimulation of intracellular cAMP and phosphorylation of CREB (cAMP response element-binding protein). Moreover, guanosine treatment increased BDNF mRNA levels suggesting the involvement of cAMP/CREB in neurotrophic effects promoted by guanosine [62].

In coculture of neuron and astrocytes pre-treated with guanosine, the inhibition of MAPK/ERK, CaMKII, PKC, PI3K or PKA blocked the reorganization of extracellular matrix proteins from a diffuse to a fibrillar matrix [145]. Furthermore, the effect of guanosine in increasing the number of cerebellar neurons cultured was also blocked by inhibition of ERK, CaMKII, PKC, PI3K and PKA [39].

Therefore, considering the neuroprotective and trophic effects promoted by guanosine, it is imperative to identify its membrane binding sites (putative receptor or selective target proteins) and understand the sequence of cell signaling pathways activation by guanosine in order to unravel the mechanisms triggered by this nucleoside. The studies discussed in this review add important information regarding the mechanism of action of guanosine, contributing for its potential use as a pharmacological strategy against neurodegenerative and neurotoxic events.

## Implications of guanosine effects towards a clinical strategy

Guanosine presents beneficial effects in several rodent and cellular models of neurodegeneration, mainly brain diseases associated to glutamatergic system unbalance, as aging-related disorders, mood-related disorders and peripheral damages that affect the CNS. Additionally, studies describe guanosine as a safe drug once there is no evidence of toxicity after exogenous guanosine administration. Recently, an intravenous guanosine administration used to evaluate vascular cardiorenal effects showed that guanosine may increase adenosine release and promote anti-inflammatory vascular effects [162]. Also in this study, guanosine *per se* has little effect on basal arterial blood pressure or renal blood flow and therefore it would be safer than adenosine for *in vivo* administrations. Nowadays, there is no effective treatment for ischemia in humans and the current available treatment, the tissue plasminogen activator (t-PA), is not effective for all patients [163]. Results obtained in ischemic models in rodents suggest an effectiveness of guanosine during the window of treatment opportunity, between the ischemia onset and irreversible neuronal death [75]. Taken together, these studies suggest guanosine as an interesting putative clinical strategy in humans. However, currently there is a lack of specific studies on guanosine toxicity and distribution after oral administration.

Recently, statins have been also prescribed after ischemia onset in order to prevent vascular damage [164]. Atorvastatin is a statin with the classical hypocholesterolemic effect and it has been shown to modulate glutamate-induced toxicity *in vivo* [165] and *in vitro* [166], similar to guanosine. Interestingly, atorvastatin treatment shows to limit the infarct size in ischemic myocardium by activating 5’-nucleotidase the enzyme that produces adenosine or guanosine [167]. An additional correlation between the effect of these two neuroprotective agents comes from the evidence that guanosine may increase cholesterol efflux from astrocytes and rat astrocytoma and increase expression of apolipoprotein E (ApoE) in astrocytes [168]. The guanosine effect of modulating cholesterol levels or distribution in cell membranes is still not clear.

Regarding purines metabolism, it has been suggested that drugs that facilitate the salvage pathway of purines recycling, as the inhibitor of xanthine oxidase activity allopurinol, may represent a clinical strategy in refractory epilepsy [169] and as an adjuvant therapy for poorly responsive schizophrenia, refractory aggressive behavior, and mania [170-173]. This mechanism of reducing purines catabolism may increase nucleosides levels contributing to the beneficial effects observed. Analysis of post-mortem PD brains showed increased urate levels, suggesting a decreased activity of purines salvage pathway in this disease. Recently, metabolomics analysis in a transgenic mice model of PD showed decreased levels of guanosine in the brain of adult transgenic mice, in a period where altered motor symptoms were observed, and a recovery of guanosine levels in aged transgenic mice [88], what might suggest a compensatory mechanism of purines metabolism.

*In vivo* human studies have shown higher levels of guanosine in patients suffering from chronic pain and it is correlated with pain severity [174]. In pregnant women levels of GTP and guanosine are increased in CSF compared to non-pregnant women and acute pain labor is negatively correlated with adenosine levels [175]. Taken together, these human studies reinforce that purines levels and metabolism modulate the organism response to injury.

The question remains whether guanosine has (or not) a selective receptor and present the necessity of developing selective pharmacological tools to study and modulate the guanine-based purinergic system. Currently, several studies are addressing the possible targets to guanosine, considering the “orphan ligand” guanosine situation. If a guanosine receptor does exist [148], it would share some (structural) features with adenosine receptors or adenosine receptors-containing oligomers [124, 155], regarding adenosine receptors antagonist and agonists effects over guanosine actions. Additionally, the guanosine interaction with potassium channels [119, 121, 124] opens a new mechanism related to the neuroprotective effect of guanosine that deserves further investigation. It is likely that guanosine may have cell-selective effects, as mediating inflammatory actions in microglia, increasing glutamate uptake in astrocytes and activating K^+^ channels in neuronal cells, therefore orchestrating an integrated cellular symphony to promote neuroprotection.

## Concluding remarks

Guanosine is an endogenous molecule that exerts neuroprotection in several disease models; however, its mechanisms of action are not well clear. Studies have proposed some guanosine receptors to explain the protective role of guanosine. However, guanosine may also act as a multi-target neuromodulator, because of its interaction with others systems, as adenosine receptors, glutamate transporters and potassium channels. It is feasible that guanosine composes an endogenous modulatory system integrating glutamatergic and purinergic transmission, gathering systems that are responsible for important plastic effects on the CNS.

Finally, mechanistic studies on guanosine action are necessary to better define the guanine-based purinergic system. These evaluations will support the future direction of clinical investigations and evaluations of safety profile that will support the use of guanosine or drugs acting at purines metabolism as clinical strategies against neurodegeneration.
